# 

**Published:** 1834

**Authors:** 


### INDEX.

Alcohol 13
Anatomy and Physiology, Popular Study of, 99
Amenorrhoea, effects of Mammary irritation on 153
Amnesia Partial case of, 209
Alkaloids the new 272
Accouchements statistics of, at the Maternite 281
Asylum Lunatic of Ohio 305
Abdominal disease complicated case of, 392
Acid sulphuric as a remedy for saturnine colic 595
Air its effects on the surface of the Body 600
Aphthous sore mouths in adults 638
Botanical specimens 18
Breasts swollen, Ranque's remedy for, 119, 283
Brain, wounds of the, 212
Do. and of the Longitudinal sinus, 319
Belladonna in Rigidity of the os uteri—in parturtion, 282
Do. in Pertussis, 292
Bath hot and cold in alternation, 317
Backwoodsman in Philadelphia, 328, 488
Blood, the influence of Gravity upon, &c. 427
Cholera, Epidemic in Indiana, 9
Do. do. Campeache, 324
Do. do. Butler co. O. 326
Catheter and sound, 158
Cholera Epidemic and Dysentery, Historical and Aetiological remarks, 169
Time of the disease, 175
Cholerine, 175
its relation to endemic Cholera, 176
treatment of, 176
miscellaneous facts and cases, connected with the disease, 185
Consumption Pulmonary, 216
Cotton in Surgical cases, 284
Classes of the Lexington and Cincinnati Medical schools, 312
Convention Medical, at Columbus, remarks on, 482
parting song of, 485
Cathartics, 557
Cyclopaedia of practical Medicine, 575
Colchicum autumnale in leurcorrhoea 594
Creosote, 595
New method of preparing, 598
medical properties of, 598
Delirium Tremens, 43
morbid anatomy of brain in, 49
stomach, 49
liver, 50
anomalous morbid appearances, 50
Emetics in, 53
Cathartics in, 54
Opium in, 55
Cold affusion, 60
Ardent spirits, 61
Moral Treatment of, 61
Datura stramonium case of poisoning by, 165
Dysentery in combination with cholera, 176
cause of the combination, 177
treatment of, 184
Dysentery and other diseases, 194, 446
Digitalis purpurea, properties and effects of the, 409
Diarrhoea, treatment of the, 417
Duodenitis, 436
Epsom salts, improved method of administering, 127
Eye operative, surgery of, 155
Extraction of cataract from, 616
Ergot—virtues of and proximate elements of, 284
Ears dry with partial deafness, treatment of, 285, 594
Electricity, present state of knowledge of, 298
Ethics Medical, 478
Ear reunion of, after complete separation, 601
Erysipelas mercurial inunction in, 609
Effusion of Blood into the pericardium fatal, 611
Fœtus, The advantage of turning by the head rather than by the feet. 141
Injuries of the head of in passing thro' the Pelvis, 282
Femur, Fracture of the neck 144
Bony union of, 269
Follicular Enterities, 254
Fractures ununited resection of Bones in, 267
successfully Treated, 293
Fractured limbs, treatment of, 268
Gonorrhoea, secale cornutum in, 110
and syphilis identity of, 602
Galvanometer, Dr. Locke's, 201
discoid description of, 202

Galvanic apparatus, 644
Gastritis chronic, 256
treatment of, 260
Herpes, 119
Hemiplegia facial, external use of Phosphorus in, 126
Hospitals, national, 301
Commercial and Lunatic Asylum of Ohio, ticket of admission to, 310
Heart, experiment upon the sounds of, 424—585
Hearing organ of, the relation of the cranium to, 425
Humerus—method of reducing the head of, in dislocation, 615
Iron, fresh prepared carbonate of, 119
Iodine in mercurial salivation, 129
in Hydrocele, 267
effects of, 277
Ileitis, treatment of, 410
Ileum inflammation of, 437
in children, 441
Intestines large diseases of, 446
Inhalations in consumption, 602
Inverted toenail, 636
Juice Gastric, experiments and observations on, 77
Jejunum inflamation of, 437
Library of Med. College of Ohio, 308
Liver anatomy and physiology of, 108
Leucorrhœa secale cornutum in, 109
Local diseases remarks on, 123
Locust, 160
Lymphatic vessels—memoir on, 250
Lithotomy, 272
Lead acetate of in disease of the mucous membranes, 287
in cholera, 402
Life animal, 486
Leeches—preservation of, 599
Medicinal plants cultivation of in Ohio, 156
Medical convention, 156—323—453
Medical men, Longevity of, 275
Meteors, 296
Meteorological notice, 321
table, 322
Man, moral and intellectual energies of, 375, 486
Medical College of Ohio 488, 638
Medical Journals 637
Medical Graduations 644
Medical Society of Cincinnati 645
Nerves spinal, functions of anterior and posterior roots of, 105
Clinical proofs of, 316
Neuralgias, remarks on the nature and treatment of, 130—293
Nitrate of Silver, in treatment of sore nipples, 594
Orchitis Gonorrhœal seat and nature of, 409
Oil croton external use of, 447
Obituary notice, 646
Porrigo ointment for, 127
Pomatum for the hair, 127
Publisher notice of, 155
Proper names, case of loss of memory for, 209
Potash Hydriodate of, in dropsy 288
Professional underbidding, 289
Pox Small, 487
Pulmonary consumption, effects of a change of climate on, 163—624
Rectum stricture of the, 138
Rheumatism treatment of, 282
Rattlesnake bite cured by the viola ovata, 288
Rice the cause of cholera, 295
Revaccinnation, 590
Specimens Botanical, 18
directions for placing in port folio, 22
times for procuring, 27
directions for drying, 29
dried, directions for arranging, 36
Somnambulism, 62
Stomach, Hypertrophy of muscular coat of, 122
cancer of the, 433
Pump—White's improved, and cupping apparatus, 487
Sylva Americana, 238
Sympathy between the mammæ and uterus, 249
Skin structure and functions of, 426
Secretions chemical properties of, 432
Strychnine in Paralysis, 625
Scrophula and Tubercular consumption, 642
Sassaparilla compound extract of, 644
Summer residence for consumption, 163
Tumours sanguineous of the cranium, 137
Truss, 156—315
Trees shade, suggestions in transplanting, 246
Typhus fever pathology of, 252
Trephine, 314
Tabes mesenterica, 443
Thorax malformation of, 634
Urinary calculi in a young female, 320
University of Transylvania, 488
Ulcers of the leg, 614
Varicocele, 121
Vertebral column, 423
Variola, 487
Western Flora, 330—486
Willoughby, medical school, 643
Yellow Fever, 628

